# *Lactobacillus rhamnosus* and *Lactobacillus casei* Affect Various Stages of *Gardnerella* Species Biofilm Formation

**DOI:** 10.3389/fcimb.2021.568178

**Published:** 2021-02-19

**Authors:** Yuanhui He, Risu Na, Xiaoxi Niu, Bingbing Xiao, Huixia Yang

**Affiliations:** Department of Obstetrics and Gynecology, Peking University First Hospital, Beijing, China

**Keywords:** *Lactobacillus rhamnosus*, *Lactobacillus casei*, biofilm formation, bacterial vaginosis, *Gardnerella* species

## Abstract

Bacterial vaginosis (BV) and its recurrence are most commonly associated with the formation of *Gardnerella* species biofilm. Probiotics are typically used to treat BV; however, the optimal period of *Lactobacillus* probiotic application in BV treatment remains uncertain. The present study aimed to explore the effects of *Lactobacillus rhamnosus* and *Lactobacillus casei* on various stages of biofilm formation in *Gardnerella* species. The biofilm-forming ability of seven strains, including one *Gardnerella vaginalis* ATCC 14018 and six clinically isolated *Gardnerella* species, was determined *via* gentian violet staining assay. Moreover, the sensitivity of the planktonic and biofilm forms toward metronidazole and clindamycin was assessed *via* microdilution broth method. *L. rhamnosus* Xbb-LR-1 and *L. casei* Xbb-LC-1 were added during various stages of biofilm formation in *Gardnerella* species and were cocultured for 24 h. The biofilm thickness of each sample was determined *via* confocal laser scanning microscopy (CLSM). The absolute quantities of *Gardnerella* species in each sample was obtained *via* real time polymerase chain reaction method, and the pH value was obtained using a pH indicator paper. Biofilm formation by *Gardnerella* species in a medium with distinct pH values was observed *via* gentian violet staining, CLSM, and scanning electron microscopy (SEM). The biofilm increased the resistance of *Gardnerella* species toward metronidazole and clindamycin. *L. rhamnosus* added at the initial biofilm formation stage in *Gardnerella* species exhibited highest inhibitory effect, with a percentage inhibition of 38.17% ± 1.35%. When the pH value of the culture medium was <4.5 or >6.5, ATCC 14018 could hardly form a biofilm; however, at pH ≥4.5 and ≤6.5, it was able to form a stronger biofilm. The amount of biofilm attained maximum value at optical density of 3.29 ± 0.28 (595 nm), pH 5.5, and at 36 h. Biofilm formation increases the resistance of *Gardnerella* species toward antibiotics. Maintaining an acidic vaginal environment with pH <4.5 and a vaginal microbiota dominated by *Lactobacillus* remarkably prevents the formation of *Gardnerella* species biofilm at the initial stage, which further has a significant impact on the treatment and prevention of biofilm-related infections.

## Introduction

Bacterial vaginosis (BV) is one of the most common lower genital tract infections found in women of reproductive age (Verstraelen and Swidsinski, 2019). It affects millions of women worldwide each year and is associated with infertility (Moragianni et al., 2019), ectopic pregnancy (Rajalakshmi and Kalaivani, 2016), sexually transmitted diseases (Abbai et al., 2016), adverse pregnancy outcomes (Subtil et al., 2018), and a higher risk of reproductive tract infections (Moragianni et al., 2019; Saweri et al., 2019). Patients with BV often present vaginal itching, increased vaginal secretions, pain, and odor (Rosca et al., 2020). The high rate of treatment failure and recurrence of BV might increase the risk of acquiring human papillomavirus (King et al., 2011) and human immunodeficiency virus (Haddad et al., 2018). BV has an adverse impact on women’s mental and physical health (Danielsson et al., 2011) and often leads to a high global burden and cost (Peebles et al., 2019).

Biofilm is a membrane-like structure comprising polysaccharide matrix, vitamins, and other components; biofilms surround a microorganism and are often characterized by a complex internal structure and channels containing nutrient cycles (Hardy et al., 2017). Biofilms play a pivotal role in BV (Swidsinski et al., 2005; Swidsinski et al., 2008; Swidsinski et al., 2010; Swidsinski et al., 2011; Javed et al., 2019; Verstraelen and Swidsinski, 2019), and can be recovered on the vaginal mucosa of BV patients after stopping the intake of antibiotics (Swidsinski et al., 2008).

*Gardnerella* species are one of the most virulent BV-related pathogens (Alves et al., 2014), and their initial adhesion initiates biofilm formation, which is considered a crucial step in biofilm formation (Castro et al., 2019) and the development of BV (Rosca et al., 2020). The failure of antibiotic treatment for BV patients may be due to the high population of *Gardnerella* species in the biofilm (Verwijs et al., 2020). Therefore, the present study aimed to demonstrate the adverse impact on antimicrobial resistance by biofilm formation.

Healthy vaginal microbes are generally dominated by lactic acid-producing *Lactobacillus* species, which create and sustain an acidic pH that inhibits the growth and adhesion of BV-associated pathogens (Rosca et al., 2020); however, BV is often characterized by a higher vaginal pH and the absence of lactobacilli (Reznichenko et al., 2020; Rosca et al., 2020). Several studies have demonstrated that lactobacilli can reduce the risk of BV and its recurrence (Xiao et al., 2016; Reznichenko et al., 2020). Numerous studies have revealed the effects of lactobacilli on biofilm formation of pathogens such as *Candida albicans* (Rossoni et al., 2018; Tan et al., 2018), *Staphylococcus aureus* (Merghni et al., 2017), and *Streptococcus mutans* (Keller et al., 2011).

Studies on the antagonizing effect of *Lactobacillus* species on *Gardnerella* species have mostly focused on changes in the population of *Gardnerella* species (Machado et al., 2013; Breshears et al., 2015) rather than directly observing their effect on the biofilms. *Lactobacillus rhamnosus* and *Lactobacillus casei* are commensal ingredients in probiotic drugs, which are commonly used in the BV treatment. These two *Lactobacillus* species colonized in the normal vaginal microbiota, which has a positive preventive effect in reducing the recurrence of BV (Xiao et al., 2019); this presumably occurs *via* mechanisms such as inhibition of *Gardnerella* species biofilms; however, the optimal application period of *Lactobacillus* probiotics in the treatment of BV remains uncertain. Therefore, we selected these two clinical isolates to explore their effects on various stages of biofilm formation in *Gardnerella* species.

## Materials and Methods

### Collection of Patients Specimens and Ethical Approval

The strains in our study included *Gardnerella* species ATCC14018, which has been identified as *G. vaginalis* (Vaneechoutte et al., 2019), six clinically isolated *Gardnerella* species strains (XB-01–XB-06), one *L. rhamnosus* strain Xbb-LR-1, and one *L. casei* strain Xbb-LC-1. Six strains of *Gardnerella* species were isolated from patients with BV between January and February 2020. The BV patients initially visited the Obstetrics and Gynecology Clinic of Peking University First Hospital and did not use antibiotics for a week prior to their visit. They were diagnosed through Nugent score and clinical symptoms and did not suffer from other urogenital tract infectious diseases. *L. rhamnosus* strain Xbb-LR-1 and *L. casei* strain Xbb-LC-1 were isolated from healthy volunteers (> 18 years old) at the health checkup clinic of the hospital between October and December 2019. These women did not suffer from any urogenital tract infections, had no sexual intercourse 1 week before admission, and did not use any antibiotics within 3 months of admission. All the samples were transported to the Microecology laboratory of the hospital and inoculated within 2 h after collection, and their corresponding clinical information was recorded. The Ethics Committee of Peking University First Hospital approved this study (V2.0/201504.20), and written informed consent was obtained from all the participants.

### Isolation, Identification, and Recovery of Bacteria

The culture was screened by streaking a vaginal swab in Casman blood agar medium with 5% (w/v) defibrinated rabbit blood, followed by trilinear method and further incubated at 37°C under anaerobic condition (5% CO2, 95% nitrogen) for 48–72 h (Bohr et al., 2020). Suspicious colonies were transferred to blood agar medium for 48–72 h for purification culture using the Gram’s method and observed under an optical microscope with oil immersion lens (Leica Microsystems, Mannheim, Germany). *Gardnerella* species revealed uniform morphology of short rod-shaped bacteria, whereas *Lactobacillus* presented a uniform morphology of gram-positive, large rod-shaped bacteria. After several purification cultures, the DNA from a single colony was extracted using the QIAamp DNA Mini Kit (QIAGEN, Germany), as previously described (He et al., 2020b). Briefly, the bacterial suspension was made from the sample using supplemented brain–heart infusion (sBHI) medium. SBHI comprised 9.25% (wt/v) BHI (Liofilchem), 0.3% (wt/v) glucose (Liofilchem), and 0.3% (w/v) soluble starch (Thermo Fisher Scientific, Lenexa, KS, USA). Thereafter, the samples were centrifuged at 10,000 rpm (∼11,500 × g) for 1 min. The supernatant was discarded and liquid Amies buffer GA was mixed. The proteinase K and the liquid Amies buffer GB were subsequently added to the suspension and incubated at 70°C for 10 min. Subsequently, 100% ethanol (w/v) was added followed by centrifugation. DNA was then isolated following a series of centrifugations with TIANGEN spin columns and buffer solutions. Next, DNA was eluted in 100 μl elution buffer (TE Buffer). Its quantity and quality were assessed at absorbances of 260 nm and at A260/280 nm *via* a Spectrophotometer (Nano Drop One, Thermo Fisher). Furthermore, the samples were stored at −20°C. To identify the isolates, the 16S rDNA sequences primers 27F (5′-AGAGTTTGATCCTGGCTCAG-3′) and 1492R (5′-GGTTACCTTGTTAGACTT-3′) were used (Chervinets et al., 2018). The isolates were confirmed by comparing the 16S rDNA sequences with the GenBank library using the BLAST program (https://blast.ncbi.nlm.nih.gov).

The clinically isolated *Gardnerella* species could not be identified at the species level by 16S rDNA sequencing, and hence, they were collectively named as *Gardnerella* species (Vaneechoutte et al., 2019). The strains were stored at −80°C in Microbank microbial storage tubes (PL.170/M, PRO-LAB, Canada). Every strain to be tested was recovered and purified before the experiment to ensure bacterial viability and purity. The viability of bacteria was determined by the growth and presence of bacterial colonies, whereas the purity was evaluated based on the morphology of the colony and bacteria under the oil microscope. *Gardnerella* species were grown in Casman blood agar medium with 5% (w/v) defibrinated rabbit blood, whereas *Lactobacillus* was cultivated on de Man, Rogosa, and Sharpe (MRS) broth agar (HaiBo, Qingdao, China) at 37°C under anaerobic conditions.

### Selection of the Most Suitable Medium for *Gardnerella* Species Biofilm Formation

A single colony of ATCC 14018 was picked from the Casman blood agar medium to adjust the bacterial suspension to 0.5 McFarland using a nephelometer (Biomerieux, France) with a concentration of 10^8^ CFU/ml (OD 590 = 0.5) (Turovskiy et al., 2012) in ATCC medium 1685 (M1486-02, ELITE-MEDIA, USA) and supplemented brain–heart infusion broth (sBHI) (AOBOX, Beijing, China). ATCC medium 1685 comprised 0.4% (wt/v) 4-(2-hydroxyethyl)-1-piperazineethanesulfonic acid (HEPES) (Liofilchem), 1.5% (wt/v) Proteose Peptone No.3 (Liofilchem), 0.5% (w/v) NaCl (Liofilchem), 0.5% (w/v) glucose (Liofilchem).The two culture media were used to dilute the bacterial suspension to 10^6^ CFU/ml. Two bacteria-free media served as their respective control groups; 200 μl suspension of ATCC14018 was inoculated in each well of a 96-well plate (Falcon, Corning Inc., Corning, NY). Under atmospheric conditions of 101.325 kPa, the media were diluted and further inoculated with the sample within 15 min. The biofilm formation was assessed by gentian violet staining assay (as described below) after incubation at 37°C under anaerobic conditions (Bohr et al., 2020) without agitation for 48 h.

### Quantification of Biofilm Formation by Gentian Violet Staining Assay

To quantitatively measure biofilm formation, a gentian violet staining assay was performed, as previously described (Harwich et al., 2010; Patterson et al., 2010; Castro et al., 2015) with some adjustments. Planktonic bacteria were eliminated from the wells by removing the spent medium and washing the wells with 300 μl of 1× phosphate buffer saline (PBS) (BL302A, Biosharp, China). The biofilm and bacteria adhered to the wells were retained on the wells. The wells were air-dried for 60 min, and the biofilms were stained with 200 μl 0.4% (w/v) gentian violet stain (G1070, Solarbio, Beijing, China) for 30 min. Thereafter, the wells were washed gently with 200 μl PBS to remove the excess stain and then air-dried for 5 min. The gentian violet was solubilized with 200 μl 33% (v/v) acetic acid per well. For quantitative results, the absorbance of gentian violet at a wavelength of 595 nm using an Epoch microplate spectrophotometer was determined (BioTek Instruments, Winooski, VT, USA) at 25°C. The growth of ATCC14018 biofilm in different situations was categorized according to the OD. OD cut-off value (ODc) was defined as three standard deviations (SD) above the mean OD of the negative control: ODc = average OD of negative control + (3 × SD of negative control), OD ≤ ODc = nonbiofilm producer; ODc < OD ≤ 2 × ODc = weak biofilm producer; 2 × ODc < OD ≤ 4 × ODc = moderate biofilm producer; 4 × ODc < OD = strong biofilm producer (Stepanovic et al., 2000; Stepanovic et al., 2007). The biofilm formation ability of the six clinically isolated *Gardnerella* species strains and the ATCC14018 strain was determined *via* gentian violet staining assay. All analyses were performed thrice over 3 days, with each sample in quintuplicate for technical replicates, and the results are presented as the mean ± standard deviation (SD).

### Biofilm Formation of *Gardnerella* Species at 24 and 48 h

SBHI was used in the subsequent steps as it is more beneficial for biofilm formation. A single colony was picked from the solid medium to adjust the bacterial suspension concentration to 10^8^ CFU/ml (OD 590 nm = 0.5) in sBHI (Turovskiy et al., 2012). The clinically isolated bacteria and the ATCC14018 strain were diluted to 10^6^ CFU/ml and then inoculated in 96-well plates (with 200 μl per well). Inoculation was completed within 15 min, and the strains were cultured in an anaerobic environment for 24 and 48 h, respectively. Each sample was assessed in quintuplicate for technical replicates in each assay and in three different assays for biological replicates.

### Observing Biofilm Formation *via* Confocal Laser Scanning Microscopy

CLSM was used to observe the biofilm formation of *Gardnerella* species before and after *Lactobacillus* interference and in a medium with different pH values. Biofilm staining was performed according to the manufacturer’s instructions mentioned on the Filmtracer Live/Dead biofilm viability kit (L10316, Thermo Fisher Scientific, USA) with some adjustments. Briefly, the samples were cultured in 24-well plates (Falcon, Corning Inc., Corning, NY) with a 14 mm ×14 mm circular cover glass (MutoPure Chemicals Co. Ltd) at the bottom of each well at 37°C under anaerobic conditions. The coverslip was removed after their incubation, washed thrice with sBHI, and then incubated for 25 min with 300 μl of fluorescent stain in the dark at 25°C. The stain solution was prepared with 3 μl of propidium iodide stain and 3 μl of SYTO^®^9 stain to 1 ml of sBHI. Thereafter, the samples were washed gently with sBHI thrice after incubation in the stain solution to remove the excess stain. The reactor coupon was placed upside down in a 20 × 20 mm dish and the dish was filled with approximately 1000 μl sBHI to cover the coupon surface by 2 mm, followed by observation on CLSM (LSM510, Carl Zeiss, Thornwood, NY) by an oil lens at 63× magnification combined with 0.75 zoom. Spectral Borealis lasers (green, 488 nm; red, 561 nm) were used for excitation. Each sample was manipulated for similar time periods. The images were obtained with a resolution of 246.03 μm × 246.03 μm image size. The tomographic scan was performed at intervals of 1 μm in the Z-axis direction to obtain a series of images of each layer, and the three-dimensional images were combined using OLYMPUS-FLuoView.Ver.1.6b software. The biofilm thickness of each sample was recorded, and each sample was evaluated in five fields (Beaudoin et al., 2017).

### Observation of Biofilm Formation of *Gardnerella* Species by Scanning Electron Microscopy

SEM was used to observe the formation of *Gardnerella* species biofilms in a medium at different pH values. The ATCC14018 suspension was adjusted to a final concentration of 10^6^ CFU/ml in sBHI at different pH values. The samples were cultured in 24-well plates with a 14 ×14 mm circular cover glass at the bottom of each well at 37°C under anaerobic conditions without agitation for 48 h. The sBHI in each well was removed lightly, and the samples in the well were then washed once with 300 μl 1 × PBS. The planktonic bacteria of each sample were removed and the adhered biofilm was left in the wells. The samples were fixed with 3% glutaraldehyde at 4°C overnight, dehydrated using an ethanol series, and substituted with t-butyl alcohol for freeze-drying (JFD-320, JEOL, Japan). The dried specimen was coated with platinum using an auto fine coater (JFC-3000EC, JEOL, Japan) and observed using SEM (JSM- 7900F, JEOL, Japan) at 3 kV (Thellin et al., 2016; Jung et al., 2019).

### Minimum Inhibitory Concentration and Minimal Biofilm Inhibitory Concentration of *Gardnerella* Species Toward Metronidazole and Clindamycin

MIC and MBIC of six *Gardnerella* species strains and the ATCC14018 strain toward metronidazole and clindamycin was determined *via* microdilution broth method according to the CLSI guideline (Wayne, PA, USA, 2018). The final concentrations of metronidazole and clindamycin (1442009 and 100037, National Institutes for Food and Drug Control, China) applied to the planktonic bacteria were 0.125–128 mg/L and 0.0625–64 mg/L, respectively. The final concentrations of metronidazole and clindamycin applied to the biofilm form were 0.25–256 mg/L and 0.125–128 mg/L, respectively. *Bacteroides fragilis* ATCC 25285 was used as a quality control.

After culturing the *Gardnerella* species on blood plates under anaerobic conditions for 48–72 h, single colonies were picked from Casman blood agar medium to adjust the bacterial suspension concentration to 10^8^ CFU/ml (OD 590 = 0.5) (Turovskiy et al., 2012) in sBHI. The suspension (100 μl, 2 × 10^6^ CFU/ml) was mixed with different concentrations of drugs at 1:1 volume ratio, and the mixture was inoculated in 96-well plates and cultured for 48–72 h. The modulation and inoculation processes were completed within 15 min. The sensitivity of both antibiotics was classified as follows: clindamycin (sensitive: MIC ≤ 2 mg/L; intermediate: MIC = 4 mg/L; resistance: MIC ≥ 8 mg/L), metronidazole (sensitive: MIC ≤ 8 mg/L; intermediate: MIC = 16 mg/L, resistance: MIC ≥ 32 mg/L). The breakpoints were obtained according to CLSI guideline (Wayne, PA, USA, 2018).

Briefly, 200 μl of each *Gardnerella* species suspension at a concentration of 1 × 10^6^ CFU/ml in sBHI was incubated in 96-well microplates at 37°C under anaerobic conditions without agitation for 24 and 48 h. The sBHI was removed and washed once with PBS, and the non-adherent bacteria were removed. Series of two-fold dilutions of metronidazole (0.25–256 mg/L) and clindamycin (0.125–128 mg/L) were added. After the addition of antibiotics, the biofilm was cultured under anaerobic conditions for 48 h, and MBIC was observed as previously described (Peeters et al., 2008). SBHI medium with each bacterial suspension in a 96-well plate without antibiotics was used as a growth control for each strain.

### Effects of *Lactobacillus* on Different Stages of Biofilm Formation of *Gardnerella* Species

The suspensions of ATCC14018, *L. rhamnosus* Xbb-LR-1, and *L. casei* Xbb-LC-1 with an adjusted concentration of 10^6^ CFU/ml were used in the following groups and incubated at 37°C under anaerobic conditions without agitation in 24 well plates. A 14 ×14 mm circular cover glass was placed at the bottom of each well.

As shown in **Supplementary Figure 1**. We divided the samples into three groups according to the processing procedure. Group one (in the competition assays): The control group (sample A) was a 300 μl suspension of *Gardnerella* species cultured for 24 h. Next, 150 μl suspension of *Lactobacillus* and 150 μl of *Gardnerella* species were mixed and cultured for 24 h (sample B and C). Group two (in the interference assays): The control group involved culturing of 150 μl of *Gardnerella* species suspension alone in one well for 24 h, followed by the addition of 150 μl of sBHI to the same well and culturing for 24 h (sample D). Thereafter, 150 μl suspension of *Gardnerella* species was added to the well and cultured for 24 h. Next, 150 μl of the *Lactobacillus* suspension was mixed and cocultured for another 24 h (sample E and F). Group three (in the interference assays): The control group involved culturing 150 μl of *Gardnerella* species suspension alone for 48 h, followed by the addition of 150 μl of sBHI and culturing for 24 h (sample G). Next, 150 μl suspension of *Gardnerella* species was cultured alone for 48 h, followed by the addition of 150 μl of *Lactobacillus* suspension and coculturing for another 24 h (sample H and I).

The biofilm thickness of the samples was examined by CLSM. The absolute quantities of *Gardnerella* species of the samples was performed *via* real-time PCR method as described below. Their pH values were tested by pH test paper (Jianjun, China). The last pH value was determined in the first 15 s after the bacterial suspension made contact with the test paper, and the results were confirmed at least thrice.

### Real-Time-PCR for Absolute Quantification of *Gardnerella* Species in Different Samples

Approximately 2 ml *Gardnerella* species suspension was used to extract DNA as before mentioned after it was adjusted to a concentration of 10^8^ CFU/ml in sBHI. The DNA was dissolved in a 100 μl TE solution. The original DNA concentration was set as 10^8^ copies/ml. After 10 folds serial dilution, seven DNA concentrations of 10^8^–10^2^ copies/ml were obtained, respectively. DdH2O was selected as a blank control. Real time PCR was performed with a reaction volume of 20 μl containing SYBR Green PCR Master Mix (Toyobo, Osaka, Japan), 25 nM of the primers, and 1 μl of DNA extracted from each concentration. Gv-F/R was used as primers for the specificity reference genes of *Gardnerella* species. The specificity of the primers (GV-F/R: 5′-CCGAATTTGCGATTTCTTCT-3′/5′-CGTACGGAAGTTTTGGAAGC-3′) (Castro et al., 2019) used in this study was confirmed by polymerase chain reaction (PCR) as previously described (Byun et al., 2004). The reaction conditions were as follows: 95°C for 60 s, followed by 40 cycles of 95°C for 15 s, 60°C for 15 s, and 72° C for 45 s (Byun et al., 2004). Each concentration was conducted in triplicate for technical replicates in each assay and in two assays. Ct values obtained at each concentration are expressed as the mean ± SD; a standard regression curve (concentration-CT value) was established (Lv et al., 2019). DNA was isolated from the samples of Group A, B and C, following exanimated *via* real-time PCR method. DdH2O was selected as a blank control and the DNA extracted from *L. rhamnosus*, and *L. casei* were selected as negative control. Each sample was tested in triplicate, and the mean values were calculated. The average DNA concentration (copies/ml) of each sample was then obtained and are presented as the mean ± SD.

### Effects of Different pH on the Biofilm Formation of *Gardnerella* Species

The pH of sBHI was adjusted to 3.5, 4.0, 4.5, 5.0, 5.5, 6.0, 6.5, 7.0, and 7.5. The ATCC14018 strain was used in this step. NaOH solution (10 mol/L) was prepared from NaOH particles (Thermo Fisher Scientific, USA) under aseptic conditions, followed by autoclave sterilization. Subsequently, 10 moL/L NaOH solution and 36.5%–38.0% concentrated hydrochloric acid (H1758 MSDS, Sigma-Aldrich) were used for adjusting sBHI to different pH values.

The pH value of sBHI was 6.5 as evaluated by the pH test paper test. Considering the pH value of sBHI as the initial pH value (6.5), approximately 10 μl of concentrated hydrochloric acid was added for every 0.5 reduction in pH value, and 5–10 μl of 10 moL/L NaOH solution was added for every 0.5 increase in pH value.

*Gardnerella* species suspension was adjusted to a concentration of 1 × 10^6^ CFU/ml by the corresponding medium at different pH values. Furthermore, the bacterial solution (150 μl) in each pH medium was mixed with 150 μl of sBHI at the corresponding pH value.

The resulting solution was added to the 96-well microplate and coincubated at 37°C under anaerobic condition without agitation. Their biofilms were quantified with gentian violet staining at 12 h, 24 h, 36 h, 48 h, 60 h, and 72 h during culture. The sBHI solution without bacterial suspension at 12 h, 24 h, 36 h, 48 h, 60 h, and 72 h was set as the corresponding blank control. This experiment was repeated thrice in five copies.

A 150 μl suspension at a concentration of 1 × 10^6^ CFU/ml of *Gardnerella* species was mixed with 150 μl sBHI at the corresponding pH value. Thereafter, the samples were incubated for 48 h in anaerobic wells in a 24-well plate with circular cover glass. Biofilm formation was observed by CLSM and SEM.

### Statistical Analysis

SPSS 20.0 was used as the statistical software. A paired-samples t-test was used to analyze the effect of *Gardnerella* species biofilm formation on the susceptibility of bacteria to antibiotics. Spearman analysis was used to analyze the linear correlation between the biofilm formation of *Gardnerella* species and the MICs of metronidazole and clindamycin against the planktonic and biofilm forms of the bacteria. An independent sample T test was used to analyze the difference in biofilm thickness of *Gardnerella* species after interference by different *Lactobacillus* groups. One-way ANOVA was performed to analyze the differences in biofilm thickness among the three groups. Nonparametric Wilcoxon test was used to analyze the statistical significance in the percentage of biofilms inhibited by lactobacilli added at three different periods. Variance analysis of repeated measurements was used to observe whether a significant difference was present in the amount of biofilm formation of ATCC14018 at different pH values. A *p* value < 0.05 indicated statistical significance.

## Results and Discussion

### Collection of the Bacteria

Six *Gardnerella* species strains were isolated from 15 BV patients. Their mean age, mean pH value, and mean Nugent score were 38.50 ± 2.06 years, 4.86 ± 0.39, and 8 ± 0, respectively. More details about their clinical information are summarized in **Supplementary Table 1**.

### Medium Selected for *Gardnerella* Species Biofilm Formation

Both media were beneficial for *Gardnerella* species biofilm formation (*p* = 0.000) *in vitro*. At 48 h, the ability of *Gardnerella* species to form biofilms in sBHI and ATCC media at OD 595 nm were 2.41 ± 0.12 and 1.73 ± 0.29, respectively. The OD 595 nm of the control groups of the two media were 0.40 ± 0.07 and 0.42 ± 0.09, respectively. Their difference was significant (*p* = 0.000). The biofilm-forming ability of *Gardnerella* species in sBHI was stronger than that of ATCC medium. Therefore, the sBHI medium was selected as the culture medium for *Gardnerella* species in subsequent experiments.

### Biofilm Formation Increases *Gardnerella* Species Resistance to Antibiotics

As summarized in **Table 1**, the MIC ranges of metronidazole and clindamycin against *Bacteroides fragilis* ATCC 25285 were 0.25–1 mg/L and 0.5–2 mg/L, respectively, which indicate that the experimental conditions are satisfactory and the results are credible.

**Table 1:** *Gardnerella* species biofilm formation and minimum inhibitory concentration (MIC) of metronidazole and clindamycin against the planktonic and biofilm forms.

Strains	Biofilm formation at 24 h	Biofilm formation at 48 h	Metronidazole			Clindamycin		
			MIC (mg/L)	Ratio of MBIC/MICat 24 h	Ratio of MBIC/MICat 48 h	MIC (mg/L)	Ratio of MBIC/MICat 24 h	Ratio of MBIC/MICat 48 h
XB-06	0.39 ± 0.03 (N)	1.65 ± 0.17 (M)	128	2 (256/128)	2 (256/128)	0.0625	2 (0.125/0.0625)	2048 (128/0.0625)
XB-05	0.34 ± 0.04 (N)	1.73 ± 0.18 (M)	1	4 (4/1)	16 (16/1)	0.0625	2 (0.125/0.0625)	2 (0.125/0.0625)
XB-04	0.61 ± 0.07 (W)	3.23 ± 0.61 (S)	16	8 (128/16)	16 (256/16)	0.0625	4 (0.25/0.0625)	2048 (128/0.0625)
XB-03	0.35 ± 0.04 (N)	1.81 ± 0.33 (M)	0.125	8 (2/0.125)	8 (2/0.125)	0.0625	2 (0.125/0.0625)	2 (0.125/0.0625)
XB-02	0.34 ± 0.03 (N)	2.20 ± 0.60 (M)	0.125	1 (0.125/0.125)	8 (1/0.125)	0.0625	2 (0.125/0.0625)	2 (0.125/0.0625)
XB-01	0.37 ± 0.03 (N)	2.50 ± 0.40 (M)	2	4 (8/2)	4 (8/2)	0.0625	2 (0.125/0.0625)	2 (0.125/0.0625)
ATCC14018	0.97 ± 0.13 (M)	2.32 ± 0.19 (M)	16	4 (64/16)	4 (64/16)	0.0625	16 (1/0.0625)	512 (32/0.0625)

N, no biofilm producer; W, weak biofilm producer; M, moderate biofilm producer; S strong biofilm producer. The biofilm formation capacity at 48 h positively correlated with both ratios of MBIC/MIC in metronidazole (correlation coefficient = 0.601, p = 0.018) and the ratio of MBIC/MIC in clindamycin (correlation coefficient = 0.318, p = 0.000) at 48 h. The biofilm formation capacity at 24 h positively correlated with the ratio of MBIC/MIC in clindamycin at 24 h (correlation coefficient = 0.949, p = 0.000). No correlation was observed between the biofilm formation capacity at 24 h and the ratio of MBIC/MIC in metronidazole at 24 h (correlation coefficient = 0.350, p > 0.05).

The biofilm acts as a barrier and protects the enclosed bacterial cells against the principal antibiotic therapy, which often results in treatment failure and persistent infection (Verstraelen and Swidsinski, 2019; Rosca et al., 2020). The biofilm formation is divided into several stages of bacterial aggregation, maturation, and dispersion (Muzny et al., 2019). It has been demonstrated that the biofilm can increases the resistance of *Gardnerella* species to both metronidazole and clindamycin. The biofilm formation capacity at 48 h positively correlated with both ratios of MBIC/MIC in metronidazole (correlation coefficient = 0.601, *p* = 0.018) and the ratio of MBIC/MIC in clindamycin (correlation coefficient = 0.318, *p* = 0.000) at 48 h. The biofilm formation capacity at 24 h positively correlated with the ratio of MBIC/MIC in clindamycin at 24 h (correlation coefficient = 0.949, *p* = 0.000). No correlation was observed between the biofilm formation capacity at 24 h and the ratio of MBIC/MIC in metronidazole at 24 h (correlation coefficient = 0.350, *p* > 0.05). However, numerous strains are required for further validation. Moreover, none of the official agencies, that is, EUCAST or CLSI, have set up standardized definitions of biofilm endpoint parameters (Thieme et al., 2019). Changes in drug sensitivity after the formation of biofilms in the present study were determined by fold point changes observed *via* gentian violet staining assay (Peeters et al., 2008), and more rigorous judgment methods will be adopted in subsequent studies.

Biofilm formation increased the antibiotic resistance of *Gardnerella* species presumably through the following mechanisms. (1) The bacteria in the biofilm core consumed the oxygen and nutrients, which slowed down the growth and metabolism of bacteria in the inner stratum of the biofilm. When exposed to antibiotics, the bacteria acquired increased tolerance, with reduced susceptibility and without undergoing genetic changes (Prax and Bertram, 2014). (2) Relatively large antibiotic compounds were restricted by the matrix, which slowed down penetration through the biofilm (Cerca et al., 2005; Jefferson et al., 2005). (3) The matrix components neutralized the effects of the antibiotics (Fux et al., 2005). (4) The applied antibiotics were degraded by live bacteria after they were pumped out of the biofilm (Van Acker et al., 2014). Due to these mechanisms, the biofilm-associated infections generally manifest as persistent chronic progressive infections and are characterized by relapses.

The treatment of biofilm-related diseases with antibiotics is associated with several challenges. New treatment programs are constantly being explored, and the use of probiotics to restore the vaginal environment is one of the present BV treatment programs (Kim et al., 2019; Reznichenko et al., 2020; Yang et al., 2020). Biofilm formation generally includes four stages: adhesion, microcolony formation and coaggregation, maturation, and dispersion (Muzny et al., 2019). Therefore, the treatment direction should not focus solely on formed biofilms but also on different growth stages of biofilms (Lamret et al., 2020). Therefore, this study attempted to use two different clinically isolated *Lactobacillus* species (Xbb-LR-1 and Xbb-LC-1) to interfere at the different stages of biofilm formation in *Gardnerella* species.

### Changes in Biofilm When *Lactobacillus* Compete and Interfere at Different Stages of Biofilm Formation of ATCC14018

**Figure 1:**
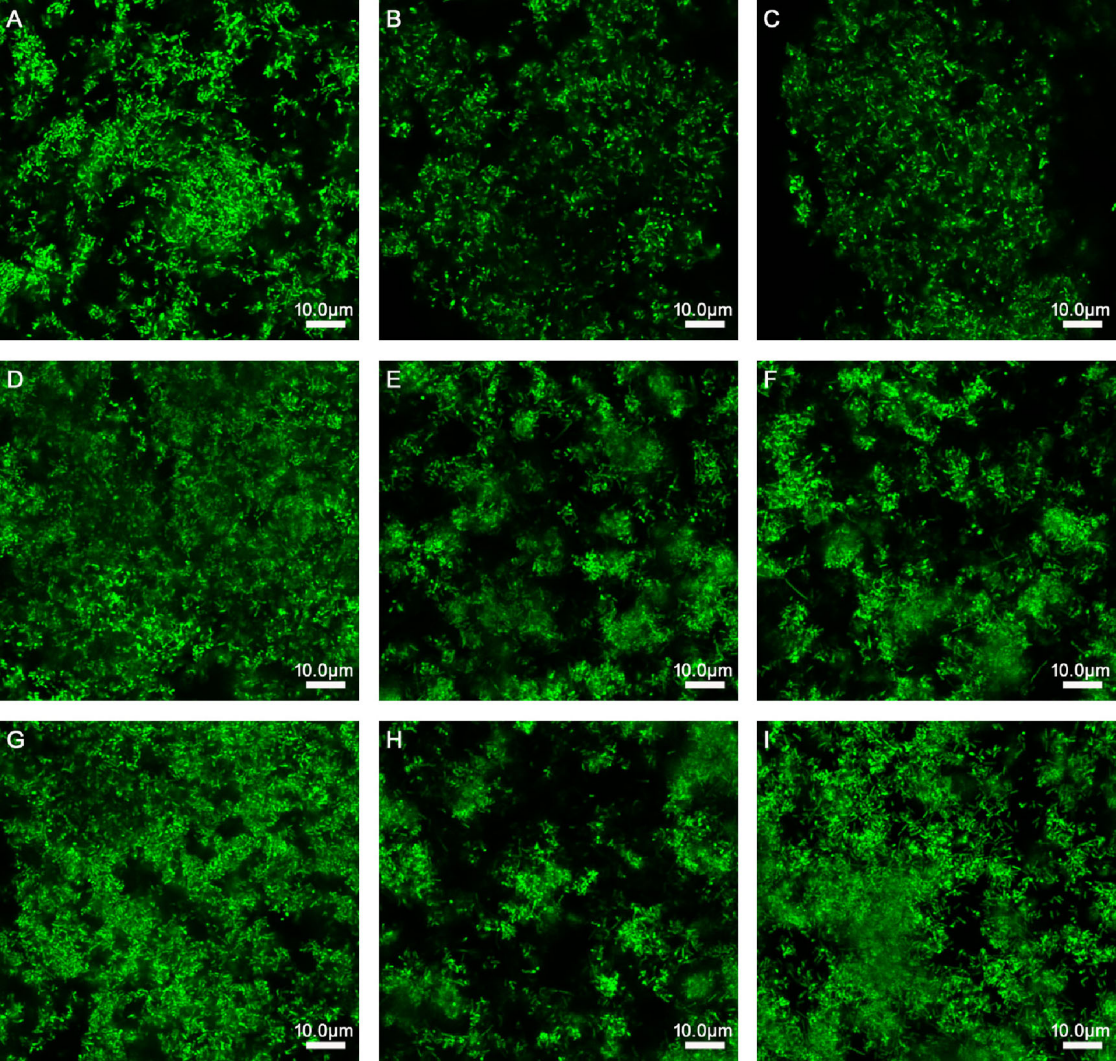
Effects of *Lactobacillus* species on different stages of biofilm formation of *Gardnerella* species. **(A)** 150 μl of *Gardnerella species* suspension and 150 μl of supplemented brain–heart infusion (sBHI) were mixed and cultured for 24 h. **(B)** 150 μl of *Gardnerella* species suspension and 150 μl of *L. rhamnosus* suspension were mixed and cultured for 24 h. **(C)** 150 μl of *Gardnerella* species suspension and 150 μl of *L. casei* suspension were mixed and cultured for 24 h. **(D)** After culturing 150 μl of *Gardnerella species* suspension alone for 24 h, 150 μl of sBHI was added and the mixture was cultured for 24 h. **(E)** After culturing 150 μl of *Gardnerella* species suspension alone for 24 h, 150 μl of *L. rhamnosus* suspension was added, followed by coculturing for 24 h. **(F)** After culturing 150 μl of *Gardnerella* species suspension alone for 24 h, 150 μl of *L. casei* suspension was added and the cells were cultured for another 24 h. **(G)** After culturing 150 μl of *Gardnerella* species suspension alone for 48 h, 150 μl of sBHI was added and the mixture was cultured for 24 h. **(H)** After culturing 150 μl of *Gardnerella* species suspension alone for 48 h, 150 μl of *L. rhamnosus* suspension was added and the mixture was cultured for another 24 h. **(I)** After culturing 150 μl of *Gardnerella species* suspension alone for 48 h, 150 μl of *L. casei* suspension was added and the mixture was cultured for 24 h. The images were captured at a magnification of 63× combined with 0.75 zoom.

As presented in **Table 2** and **Figure 1**, when *L. rhamnosus* was added to the biofilm samples formed by ATCC14018 at 0, 24, and 48 h (**Figures 1B, E, H**), the biofilm formation was significantly inhibited, with a percentage inhibition of 38.17% ± 1.35%, 32.66% ± 1.99%, and 29.43% ± 2.68%, respectively, and their *p* values were all 0.000. The interference effect of *L. rhamnosus* on the biofilm formed at 0 h of *Gardnerella* species was the strongest, with a significant difference, compared with the percentage reduction of biofilm formed at 24 and 48 h (both *p* values were 0.000).

**Table 2:** Thickness of the biofilm, absolute quantities of *Gardnerella* species/*Lactobacillu*s, and pH value after the interference of *Lactobacillus* at different stages of ATCC 14018 cultivation.

Group	Samples	Biofilm thickness (μm)	Reduction in biofilm thickness (%)	Absolute quantities of *Gardnerella* species (log copies/ml)	Reduction in quantities of *Gardnerella* species (%)	pH
Group one	**A:** 150 μl of *Gardnerella species* suspension and 150 μl of sBHI were mixed and cultured for 24 h.	36 ± 0.5^a^	–	7.26 ± 0.1^p^		6.5
	**B:** 150 μl of *Gardnerella* species suspension and 150 μl of *L. rhamnosus* suspension were mixed and cultured for 24 h.	23 ± 0.7^b^	38.17 ± 1.35^j^	5.82 ± 0.13^q^	19.88 ± 2.29^y^	4.1
	**C:** 150 μl of *Gardnerella* species suspension and 150 μl of *L. casei* suspension were mixed and cultured for 24 h.	33 ± 0.7^c^	10.88 ± 1.85^k^	6.78 ± 0.05^r^	6.70 ± 0.89^z^	4.8
Group two	**D:** After culturing 150 μl of *Gardnerella species* suspension alone for 24 h, 150 μl of sBHI was added and the mixture was cultured for 24 h.	48 ± 1.5^d^	–	7.72 ± 0.09^s^		6
	**E:** After culturing 150 μl of *Gardnerella* species suspension alone for 24 h, 150 μl of *L. rhamnosus* suspension was added, followed by coculturing for 24 h.	33 ± 0.8^e^	32.66 ± 1.99^l^	6.66 ± 0.12^t^	13.69 ± 1.83^α^	3.8
	**F:** After culturing 150 μl of *Gardnerella* species suspension alone for 24 h, 150 μl of *L. casei* suspension was added and the cells were cultured for another 24 h.	43 ± 0.9^f^	12.57 ± 0.74^m^	7.21 ± 0.11^u^	6.61 ± 1.69 ^β^	4.6
Group three	**G:** After culturing 150 μl of *Gardnerella* species suspension alone for 48 h, 150 μl of sBHI was added and the mixture was cultured for 24 h.	47± 1.5^g^	–	7.60 ± 0.08^v^		6
	**H:** After culturing 150 μl of *Gardnerella* species suspension alone for 48 h, 150 μl of *L. rhamnosus* suspension was added and the mixture was cultured for another 24 h.	33 ± 1.5^h^	29.43 ± 2.68^n^	6.99 ± 0.05^w^	7.98 ± 0.80 ^γ^	3.8
	**I:** After culturing 150 μl of *Gardnerella species* suspension alone for 48 h, 150 μl of *L. casei* suspension was added and the mixture was cultured for 24 h.	48 ± 1.0^i^	0.57 ± 1.55°	7.65 ± 0.01^x^	-^δ^	4.4
	**J:**150 μl of *L. rhamnosus* suspension and 150 μl of sBHI were mixed and cultured for 24 h.	–	–	–	–	3.8
	**K:**150 μl of *L. casei* suspension and 150 μl of sBHI were mixed and cultured for 24 h.	–	–	–	–	4.4

P_ab_ = 0.000; P_ac_ = 0.000; P_de_ = 0.000; P_df_ = 0.000; P_gh_ = 0.000; P_gi_ = 0.287; P_jl_ = 0.000; P_jn_ = 0.000; P_ln_ = 0.051; P_km_ = 0.091; P_ko_ = 0.000; P_mo_ = 0.000; P_jk_ = 0.000; P_lm_ = 0.000; P_no_ = 0.000; P_pq_ = 0.000; P_pr_ = 0.000; P_st_ = 0.000; P_sv_ = 0.000; P_vw_ = 0.000; P_vx_ = 0.582; P_ps_ = 0.000; P_pv_ = 0.001; P_yα_ = 0.000; P_αγ_ = 0.000; P_zγ_ = 0.000; P_zβ_ = 0.927; P_βδ_ = 0.001.(e: P_ab_ < 0.05 indicates statistical significance between a and b).

When *L. casei* was added to the samples of biofilm formed by *Gardnerella* species at 0, 24, and 48 h (**Figures 1C, F, I**), the biofilm thickness decreased, and the percentage reductions in biofilm formation were 10.88% ± 1.85%, 12.57% ± 0.74%, and 0.57% ± 1.55%, respectively. The *p* values were 0.000, 0.000, and 0.287, respectively. *L. casei* had little effect on biofilm formation after 48 h. The thickness reduction of biofilm formed at 48 h by *L. casei* was significantly different from that of biofilm formed at 0 or 24 h, and both *p* values were 0.000.

### The Changes in Quantification of ATCC14018 When *Lactobacillus* Compete and Interfere

A stand curve was established with y = −3.1036X + 35.048 from the serially diluted DNA copies in relation to Ct values. The linear regression slope was determined to be −3.1036 with a correlation coefficient (R^2^) of 0.999 (**Supplementary Figure 3**). The DNA concentration range from 10^8^ to 10^2^ copies/ml. The dissolution and amplification curves are illustrated in **Supplementary Figure 2**. The specificity of primers for detecting bacteria *via* PCR are illustrated in **Supplementary Figure 3**.

As summarized in **Table 2**, in the competitive assays, the quantities of *Gardnerella* species in samples A, B, C were 7.26 ± 0.1, 5.82 ± 0.13, and 6.78 ± 0.05 copies/ml, respectively. *Lactobacillus* addition at this stage revealed the strongest inhibitory effect, and the reduction in quantities of *Gardnerella* species were 19.88% ± 2.29% and 6.7% ± 0.89%, respectively. In the interference assays, when *L. rhamnosus* and *L. casei* were added after *Gardnerella* species biofilm formation at 24 h, the quantities of *Gardnerella* species were reduced by 13.69% ± 1.83% and 6.61% ± 1.69%, respectively. When *L. rhamnosus* and *L. casei* were added after *Gardnerella* species biofilm formation at 48 h, the reduction in quantities of *Gardnerella* species was not obvious.

At the initial stages of *Gardnerella* species biofilm formation, *Lactobacillus* revealed a competitive adhesion with *Gardnerella* species *via* secretion of a bactericidal substance, the adhesion competition effect, and coaggregation (Pino et al., 2019; Barzegari et al., 2020) and presented the greatest inhibitory effect on the growth of *Gardnerella* species.

The inhibition of *Gardnerella* species biofilm by *Lactobacillus* during the formation or maturation phase was not as effective as that during the initial phase. This may occur because the addition of *Lactobacillus* had an adhesion-replacement effect on *Gardnerella* species, whereas *Gardnerella* species had adhesion and rejection effects on *Lactobacillus* (He et al., 2020a). Although the presence of *Lactobacillus* can acidify the surroundings of *Gardnerella* species, the biofilm enhances the tolerance of *Gardnerella* species to lactic acid by preventing the inhibition of physical adhesion by *Lactobacillus* and delaying the penetration of bactericidal substances secreted by *Lactobacillus* (Verstraelen and Swidsinski, 2019). We hypothesized that *Gardnerella* species may simultaneously release and accumulate numerous harmful substances, thereby inhibiting the growth of *Lactobacillus*; however, further studies are needed to confirm this hypothesis.

The inhibitory effects of *L. rhamnosus* and *L. casei* on biofilm formation differed. Barzegari and colleagues reported that different strains of probiotics often present various antibacterial effects (Barzegari et al., 2020).

### Effects of Different pH on the Initial Biofilm Formation of ATCC14018

We tracked the pH value in different samples after the biofilm formation of *Gardnerella* species was affected by lactobacilli production (see **Table 2**) and speculated that the acidification environment created by *Lactobacillus* could effectively inhibit the initial formation of *Gardnerella* species biofilm. Therefore, we investigated the effects of different pH on the initial biofilm formation of *Gardnerella* species.

**Figure 2:**
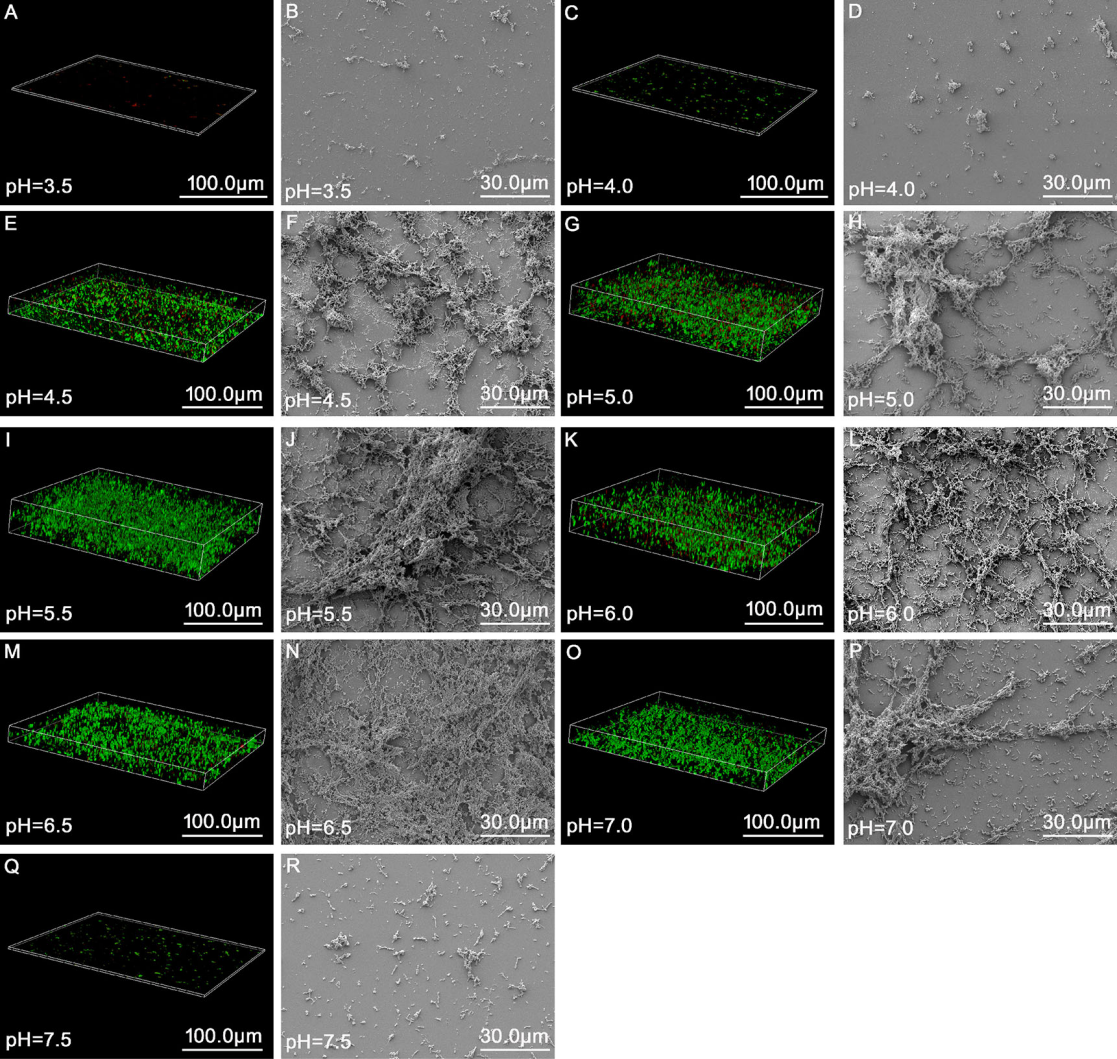
Morphology and structure of biofilms of *Gardnerella* species at up to 48 h of incubation at different pH values as observed *via* fluorescence microscopy and scanning electron microscopy. **(A, C, E, G, I, K, M, O, Q)** represent the state of biofilms observed under a fluorescence microscope (63×, zoom = 0.75, magnification) at pH 3.5, 4.0, 4.5, 5.0, 5.5, 6.0, 6.5, 7.0, and 7.5, respectively. **(B, D, F, H, J, L, N, P, R)** refer to the structure of biofilms observed *via* scanning electron microscopy (1,000× magnification) at pH 3.5, 4.0, 4.5, 5.0, 5.5, 6.0, 6.5, 7.0, 7.5, respectively.

As summarized in **Table 3** and **Figure 2**, the results of the growth curve of the biofilm obtained *via* gentian violet staining assay indicated that when the pH was 3.5 or 7.5, ATCC14018 could hardly form biofilms; when the pH was 4.0 or 7.0, *Gardnerella* species formed a weak biofilm; and when the pH ranged from 4.5 to 6.5 (particularly 5.0–6.0), strong biofilms were formed, reaching a maximum of 3.29 ± 0.28 (OD 595 nm) at pH 5.5 and at 36 h.

**Table 3:** Effects of different pH values on the biofilm formation of ATCC 14018.

pH	OD measurement at 12 h (OD595nm)	OD measurement at 24 h (OD595nm)	OD measurement at 36 h (OD595nm)	OD measurement at 48 h (OD595nm)	OD measurement at 60 h (OD595nm)	OD measurement at 72 h (OD595nm)
Blank control	0.23 ± 0.04	0.34 ± 0.02	0.38 ± 0.08	0.40 ± 0.06	0.43 ± 0.06	0.26 ± 0.05
3.5	0.46 ± 0.13 (W)	0.49 ± 0.12 (W)	0.52 ± 0.07 (N)	0.47 ± 0.04 (N)^a^	0.54 ± 0.06 (N)	0.48 ± 0.12 (W)
4	0.57 ± 0.12 (W)	0.61 ± 0.05 (W)	0.71 ± 0.11 (W)	0.67 ± 0.13 (W)^b^	0.72 ± 0.08 (W)	0.69 ± 0.10 (W)
4.5	1.18 ± 0.05 (M)	1.82 ± 0.28 (S)	2.03 ± 0.29 (S)	2.28 ± 0.25 (S)^c^	2.42 ± 0.28 (S)	2.26 ± 0.55 (S)
5	2.41 ± 0.28 (S)	2.73 ± 0.19 (S)	2.97 ± 0.51 (S)	3.06 ± 0.24 (S)^d^	3.34 ± 0.18 (S)	3.09 ± 0.18 (S)
5.5	2.69 ± 0.26 (S)	2.98 ± 0.26 (S)	3.29 ± 0.28 (S)	3.10 ± 0.25 (S)^e^	3.16 ± 0.21 (S)	3.48 ± 0.18 (S)
6	2.19 ± 0.21 (S)	2.50 ± 0.75 (S)	2.53 ± 0.53 (S)	2.98 ± 0.10 (S)^f^	3.07 ± 0.14 (S)	3.08 ± 0.42 (S)
6.5	1.07 ± 0.13 (M)	1.40 ± 0.08 (M)	1.93 ± 0.11 (M)	2.36 ± 0.26 (S)^g^	2.44 ± 0.20 (S)	2.13 ± 0.23 (S)
7	0.77 ± 0.13 (M)	0.69 ± 0.10 (W)	0.63 ± 0.12 (W)	0.71 ± 0.12 (W)^h^	0.74 ± 0.09 (W)	0.55 ± 0.09 (W)
7.5	0.48 ± 0.08 (W)	0.31 ± 0.05 (N)	0.39 ± 0.07 (N)	0.31 ± 0.04 (N)^i^	0.41 ± 0.06 (N)	0.29 ± 0.01 (N)

N, no biofilm formation; W, weak biofilm formation; M, moderate biofilm formation; S, strong biofilm formation. The results indicated that there were significant differences in biofilm formation at different pH values (p = 0.000). Intragroup comparison: Time was a significant factor for biofilm formation, and interaction was significant for biofilm formation (p = 0.000). P_ab_ = 0.203; P_bc_ = 0.000; P_cd_ = 0.000; P_de_ = 0.115; P_ef_ = 0.049; P_fg_ = 0.000; P_gh_ = 0.000; P_hi_ = 0.001.

Analysis of variance for repeated measurements was used to analyze the amount of biofilm formed by *Gardnerella* species at different pH values and growth times. When different pH and time values were used for classification between the subjects, the results revealed significant differences in biofilm formation at different pH values (*p* = 0.000). Intragroup comparison revealed that time was significant for biofilm formation, which suggests that the effect of time varies from group to group.

CLSM and SEM were combined to observe the structure of the *Gardnerella* species biofilm at 48 h at different pH. In the broth medium with a pH of 3.5 and 4.0, small scattered colonies were observed, and *Gardnerella* species could not form a mature biofilm structure (**Figures 2A–D**). When the broth medium had pH values ≥ 4.5 and ≤ 6.5, the biofilm was flocculent (**Figures 2E–N**); however, with the increase in pH value, the structure became more complex and clumped. When the pH values were 7.0 and 7.5, the formation of biofilm gradually weakened (**Figures 2O–R**). The structure of the biofilm gradually became sparse and the thickness decreased at pH 7.0 (**Figures 2O, P**). When the pH value was 7.5, only scattered small aggregates and almost no biofilm formation were observed (**Figures 2Q, R**).

Lactic acid and other organic acids produced by *Lactobacillus* change the pH of the surrounding environment of pathogens, which may be crucial in the inhibition of pathogens (Barzegari et al., 2020). The organic acids secreted by probiotics can act as quorum sensing antagonists, which can interfere with the expression of quorum sensing-related genes and prevent biofilm formation (Lopes et al., 2017).

Although pH > 6.5 may occur in the vaginal environment due to factors such as internal ejaculation or menstrual period, pH > 6.5 may not last for a long time due to the self-repairing function of the vaginal environment to maintain its balance and stability. Most patients with BV were found to have a pH value in the range of 4.5–6.0 (see **Supplementary Table 1**, more clinical data not shown). This may be because the vagina needs to undergo a change from pH > 6.5 to the range of 3.8–4.4 while returning to the normal pH range. When the pH is in the range of 4.5–6.0, the existing pathogenic *Gardnerella* species grow rapidly, and the changes in the surrounding environment promote the formation of the *Gardnerella* species biofilm. This may also be one of the reasons for relapse in BV patients.

*Gardnerella* species can hardly form biofilms when the culture medium pH is < 4.5, and they have a strong biofilm formation ability when pH ≥4.5 and ≤6.5. This confirms that acidifying the vaginal environment can reduce the biofilm production (Boskey et al., 2001). Although *Gardnerella* species can colonize the vagina of healthy women (Castro et al., 2015; Castro et al., 2020), several studies have found that the biofilm-forming ability of *Gardnerella* species from BV patients does not significantly differ from that of *Gardnerella* species from non-BV women under the same culture conditions (Castro et al., 2015; Castro and Cerca, 2015). This suggests that the clinical significance of the vaginal environment is important for biofilm formation. Therefore, maintaining an acidic vaginal environment and vaginal microbiota dominated by *Lactobacillus* are of great significance for preventing *Gardnerella* species infection and biofilm formation.

## Conclusion

This study confirms that biofilms can increase the resistance of *Gardnerella* species to antibiotics. *Lactobacillus* added during the initial stage of *Gardnerella* species biofilm formation revealed the best inhibitory effect. The acidic environment with pH < 4.5 created by *Lactobacillus* plays an important role in the formation of *Gardnerella* species biofilm at the initial stage. This finding is highly significant in the treatment and prevention of biofilm-related infections. While the evaluation of the biofilm formation capacity of *Gardnerella* species requires further improvement, the mechanism of inhibitory effect of *Lactobacillus* on *Gardnerella* species’ biofilm remains to be further explored.

## Data Availability Statement

The original contributions presented in the study are included in the article/**Supplementary Material**. Further inquiries can be directed to the corresponding authors.

## Ethics Statement

The studies involving human participants were reviewed and approved by The Ethics Committee of Peking University First Hospital (V2.0/201504.20), and written informed consent was obtained from all participants. The patients/participants provided their written informed consent to participate in this study.

## Author Contributions

YH, RN, XN, BX, and HY conceived the study design. XN and RN were responsible for the recruitment of volunteers and collection of samples. YH, RN, and XN performed the laboratory assays. YH performed the data analysis, and YH wrote the initial manuscript. BX and HY revised the manuscript. All authors contributed to the article and approved the submitted version.

## Funding

This work was supported by the National Natural Science Foundation of China (no. 81971342 and no. 81200411).

## Conflict of Interest

The authors declare that the research was conducted in the absence of any commercial or financial relationships that could be construed as a potential conflict of interest.
