# Supplementary material for: Lactobacillus rhamnosus and Lactobacillus casei Affect Various Stages of Gardnerella Species Biofilm Formation
Source: Front Cell Infect Microbiol. 2021 Feb 19;11:568178. doi: 10.3389/fcimb.2021.568178 (PMC7933028; doi:10.3389/fcimb.2021.568178)
Supplement: Supplementary file 1 [file DataSheet_1.docx]

**Supplementary Table 1.** Clinical characteristics of six isolated bacteria corresponding to BV patients

| Strain number | XB-1-01 | XB-2-01 | XB-4-01 | XB-7-01 | XB-8-01 | XB-9-01 |
| --- | --- | --- | --- | --- | --- | --- |
| Nugent score | 8 | 8 | 8 | 8 | 8 | 8 |
| WBC | <10 | <10 | <10 | <10 | <10 | <10 |
| Lactobacilli grade | III | III | III | III | III | III |
| Cell in external bottom layer | <1% | <1% | <1% | <1% | <1% | <1% |
| Clue cell | (+) | (+) | (+) | (+) | (−) | (+) |
| Amine test | (+) | (+) | (+) | (+) | (+) | (+) |
| pH value | 4.8 | 5.4 | 4.4 | 5.4 | 4.6 | 4.6 |
| Vaginal flora density | ++++ | +++ | +++ | +++ | +++ | +++ |
| Vaginal flora diversity | +++ | +++ | +++ | +++ | +++ | +++ |
| Dominant pathogens | G^-^/short rod-shaped | G^-^/short rod-shaped | G^-^/short rod-shaped | G^-^/short rod-shaped | G^-^/short rod-shaped | G^-^/short rod-shaped |
| Pruritus vulvae | No | No | Yes | Yes | No | No |
| Vulvodynia | No | No | No | No | No | No |
| Secretion amount | Abnormal | Normal | Abnormal | Abnormal | Abnormal | Abnormal |
| Vaginal rhagadia | No | No | No | No | No | No |
| Vaginal hyperemia | No | Yes | No | Yes | No | No |
| Age (years) | 39 | 35 | 38 | 39 | 42 | 38 |
| Secretion characteristics | Tenuity homogeneous | Pasty | Tenuity homogeneous; spumose | Tenuity homogeneous | Tenuity homogeneous; spumose | Tenuity homogeneous;  spumose |
| Secretion color | White | White | White | White | White | White |
| Secretion odor | Yes | Yes | Yes | Yes | Yes | Yes |
| Past medical history | VVC | VVC | BV | BV | VVC & BV | VVC & BV |
| Contraceptive method | Safe period and condom | Condom | Oral contraceptives | Intrauterine device | Condom | Intrauterine device |

No = not present. Yes = present.

**
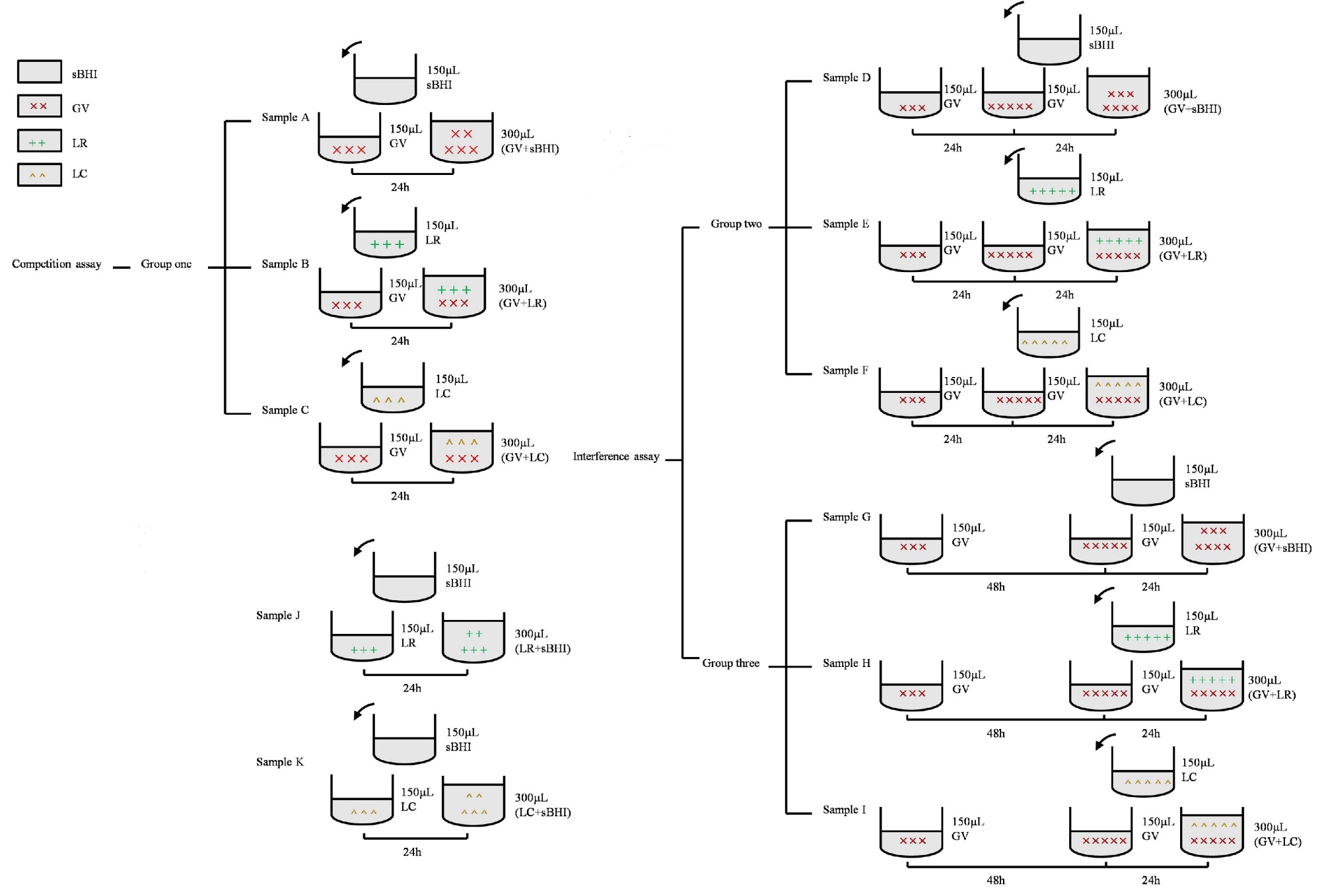
**

**Supplementary Figure 1.** GV: ATCC 14018; LR: *L. rhamnosus*; LC: *L. casei.*


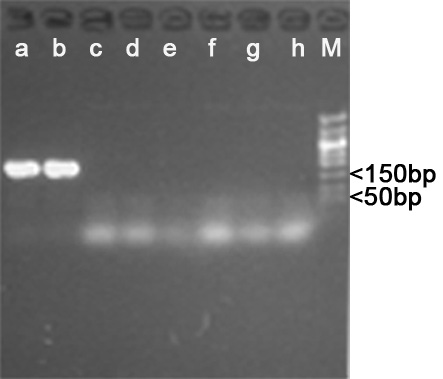


**Supplementary Figure 2.** The specificity of primers for the detection of *Gardnerella* species via polymerase chain reaction.

Lanes a and b, c and d, e and f, g and h are amplification products of DNA of *Gardnerella* species, *L. rhamnosus*, *L. casei,* and ddH_2_O by the primers of GV-F/R, respectively. M is the 50 bp DNA ladder. The lanes were obtained in 1.5% agarose gel at 90 V for 45 min.


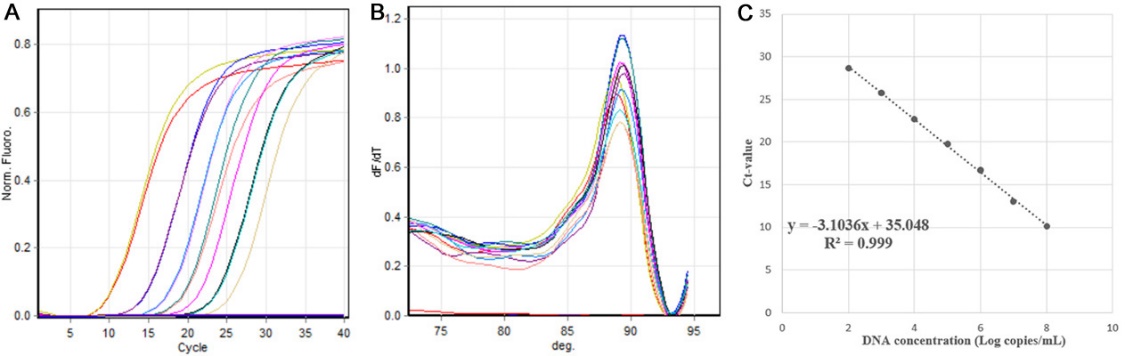


**Supplementary Figure 3.** (A) Amplification curves, (B) melting curves and (C) standard curve generated from 10-fold serial dilutions of DNA extracted from *Gardnerella* species ranging from 10^8^–10^2^ copies/mL.
